# Exploring the dielectric properties of herbal medicine and modern pharmaceuticals: an integrative review

**DOI:** 10.3389/fphar.2024.1536397

**Published:** 2025-01-15

**Authors:** Vinita Khatri, Prasanjit K. Dey

**Affiliations:** ^1^ Department of Basic Science & Humanities, Mukesh Patel School of Technology Management and Engineering, SVKM’s Narsee Monjee Institute of Management Studies (NMIMS) Deemed-to-be-University, Mumbai, India; ^2^ Department of Metallurgical Engineering and Materials Science, IIT Bombay, Mumbai, India

**Keywords:** medicine, herbal, pharmaceutical, molecular interactions, dielectric spectroscopy

## Abstract

The integration of herbal medicine with modern pharmaceuticals offers a novel approach to addressing complex healthcare challenges. This study investigates the role of dielectric spectroscopy in analysing key physicochemical properties such as solubility, stability, and molecular interactions. The findings reveal that combining herbal extracts with pharmaceutical agents enhances solubility and stability. It also reduces adverse effects, improving therapeutic efficacy. Dielectric spectroscopy is highlighted as a powerful analytical tool in this process. The study demonstrates how traditional herbal knowledge can be effectively linked with modern scientific methods. This approach enables the development of innovative therapeutic solutions that address safety and efficacy challenges. The results underline the potential of combining advanced analytical techniques with ethnopharmacological practices. This integration paves the way for the creation of safe, effective, and scientifically validated formulations for improved healthcare outcomes.

## Introduction

The foundation of traditional medicine is the extensive endeavour that humans have made to identify solutions to diseases. Natural resources laid the foundation of modern medicine now a days. Pharma industry commercially manufacturing drugs for promoting health by treating disease. About one-fourth of the drugs prescribed worldwide are derived from plants (Mohanty et al., 2017). Herbal medicines are widely used throughout the world due to their proven efficacy and easy availability. Herbal medicine is completely consisting of plentiful substances extracted from plants, homebased infusions and plants harvested for medicinal commitments.

The healthcare sector is revolutionized in the last 10 decades due to the progress and mass production of modern drugs. These drugs certainly have side effects which brings great risks to human bodies as well as pharmaceutical enterprises (Wei et al., 2024; Shi et al., 2023). The approach of combining the traditional herbal medicines with modern pharmaceuticals may overcome/minimize the side effects. Herbal drugs such as Neem and Papaya are distinguished for their anti-inflammatory, antioxidant, and immunomodulatory properties (Rombolà et al., 2020). Commonly used allopathy drug i.e., Paracetamol is a widely used analgesic and antipyretic drug. It is associated with probable hepatotoxicity at high doses or with prolonged use (Allegaert, 2020) Neem extract has shown significant liver-protective properties (Zhang et al., 2022). Similarly, papaya leaf extract is known for its ability to enhance platelet production and support liver health (Lim et al., 2021).

Integrating herbal extracts with antihepatotoxic properties may help reduce adverse effects. The interactions between modern drugs and herbal extracts are important to study. These interactions are particularly significant from pharmacokinetic and pharmacodynamic perspectives. Molecular-level studies can provide insights into optimizing dosage regimens. They can also improve treatment outcomes. Furthermore, such combinations can leverage the synergistic effects of herbal constituents. This approach enhances therapeutic efficacy while minimizing the risk of side effects. (Rombolà et al., 2020; Lee et al., 2023).

The integration of traditional herbal medicine with modern pharmaceuticals is gaining attention. It shows promise in addressing complex healthcare challenges. Ethnopharmacological approaches have traditionally focussed on biological and pharmacological evaluations. Dielectric spectroscopy is an advanced technique which offer deeper insight into molecular interactions. Analysis of physicochemical properties such as solubility, stability and dynamic molecular behaviour in herbal-pharmaceutical formulations can be precisely done using this approach (Mohanty et al., 2017; Rombolà et al., 2020). Even the formulation strategies can be optimized by studying interaction mechanisms at the molecular level. This interdisciplinary approach connects traditional ethnopharmacology with modern scientific methods. It opens new possibilities for innovative healthcare solutions.

Dielectric spectroscopy allows to study the materials in a broad range of frequency and temperature. The broad range coverage makes it a superior method as compared to calorimetry or light scattering. It provides detailed insights into both molecular relaxation processes and charge transport mechanisms (Di Noto et al., 2012). Unlike methods such as differential scanning calorimetry, this method is non-invasive and can analyze samples without altering their physical or chemical structure. This advantage makes it ideal for pharmaceutical and biological applications where sample integrity is crucial (Hosman et al., 2024). This method excels in detecting elusive relaxation dynamics, such as β-relaxation in amorphous systems, which are not easily observed through other techniques like nuclear magnetic resonance (NMR) or rheological methods. This specificity aids in studying glass transitions and molecular mobility (Romanini et al., 2023). This method uniquely bridges mechanical, thermal, and electrical properties of materials. This is the reason it is versatile for applications in condensed matter physics, nanotechnology, and material science (Deng et al., 2025).

Thus, this review aims to explore how dielectric study is useful to explore the interaction herbal extracts with modern medicines. This will lead focus on the pharmacological interactions, benefits, and potential applications. This integrative approach holds promise for more effective and safer therapeutic strategies.

## Traditional herbal medicine with modern pharmaceuticals

Herbal extracts are composed of concentrated compounds extracted from plants. They play a crucial role in medicine due to their natural therapeutic properties. The wide range of available herbal species (Table 1) includes a vast array of herbs, shrubs, trees, and other botanicals that contain medicinal compounds with potential health benefits (Hewlings and Kalman, 2017; Harty et al., 2018). In reality, approximately, 25 percent of all prescription drugs currently being utilized world over are originally derived from plants (Hostettmann et al., 1999; Walsh, 2013). Few of them are Papaya and Neem are of current interest among the vast variety of these herbal species due to their well-documented traditional uses (Singh et al., 2023; Attah et al., 2021), diverse bioactive compounds and potential therapeutic applications (Flores et al., 2024; Shanmugam et al., 2021). The bioactive molecules present in Papaya and Neem leaf extracts contribute to their various biological activities, including antioxidant, anti-inflammatory, antimicrobial, and potentially other health-promoting effects. Researchers often investigate these extracts to understand the specific bioactive compounds responsible for their beneficial properties and to explore their potential applications in medicine, agriculture, and other fields (Duhan et al., 2023; Priya et al., 2020).

**TABLE 1:** Some selected herbs among the available herbal species which are easily available and used worldwide.

Herb	Therapeutic properties	Diseases treated	Active compounds
Turmeric (Curcuma longa)	Anti-inflammatory, antioxidant	Arthritis, cancer, digestive issues	Curcumin
Ginger (Zingiber officinale)	Antiemetic, anti-inflammatory, antioxidant	Inflammation, nausea, migraines	Gingerol
Neem (Azadirachta indica)	Antibacterial, antifungal, anti-inflammatory	Skin diseases, dental disorders, infections	Azadirachtin, Nimbin
Garlic (Allium sativum)	Antimicrobial, cardioprotective, anticancer	Cardiovascular diseases, infections, cancer	Allicin
Aloe Vera (Aloe barbadensis)	Wound healing, anti-inflammatory, laxative	Skin conditions, burns, digestive issues	Aloin, Aloesin
Echinacea (Echinacea purpurea)	Immune booster, anti-inflammatory, antioxidant	Colds, flu, infections	Cichoric acid
Ginkgo (Ginkgo biloba)	Cognitive enhancer, antioxidant	Dementia, Alzheimer’s disease, circulation	Ginkgolides, Bilobalide
Ashwagandha (Winthania somnifera)	Adaptogen, anti-stress, anti-inflammatory	Stress, anxiety, inflammation	Withaferin A
Milk Thistle (Silybum marianum)	Liver protectant, antioxidant, anti-inflammatory	Liver diseases, detoxification	Silymarin
Licorice (Glycyrrhiza glabra)	Anti-inflammatory, antiviral, digestive aid	Peptic ulcers, sore throat, infections	Glycyrrhizin
St. John’s Wort (*Hypericum perforatum*)	Antidepressant, anti-inflammatory, antiviral	Depression, anxiety, infections	Hypericin
Valerian (Valeriana officinalis)	Sedative, anxiolytic, muscle relaxant	Insomnia, anxiety, muscle spasms	Valerenic acid
Peppermint (Mentha piperita)	Antispasmodic, digestive aid, antimicrobial	Irritable bowel syndrome (IBS), colds, headaches	Menthol

Allopathic medicine is the most preferred choice of medication in the world today since it is basically a drug-oriented methodology that relies on three things, hypothesis, experimentation, and results (Merriam and Mohamad, 2013). An understanding of reactions between herbal ingredients and other drugs that could either be herbal medicines and/or allopathic medicines is of particular importance when serious and/or life-threatening diseases are being treated such as rheumatic diseases, geriatric diseases, hypertensions, cancers, and AIDS that essentially require multi-drug therapies (Wagner and Ulrich-Merzenich, 2009). In Western countries, for multifactorial or complex disease treatment (e.g., cancer, hypertension, metabolic and inflammatory diseases, acquired immune deficiency syndrome (AIDS) and infections), an effective multidrug therapy is commonly adopted (Katselou et al., 2014). The herbal extracts are gaining popularity across the entire world. This popularity is expected to increase in the next few years. Consequently, the risk of herbal extracts–drug interaction remains challenging. A critical approach to herbal–drug interactions, adverse effects, and clinical efficacy should be always borne in mind when recommending or taking botanical products, with special attention on serious adverse events (Izzo et al., 2016).

Although modern medicine has been the most conventional system of medicine over the years, people are now diverting towards the utilization of herbal medicine. Modern treatments often provide only symptomatic relief rather than addressing underlying causes, are associated with serious and frustrating side effects, and are frequently expensive, making them less accessible for many individuals. These factors drive the increasing appeal of herbal alternatives, which are perceived as more natural and cost-effective (Kumar and Rai, 2011; Jawla et al., 2009).

Herbal medicine is often preferred because it is less expensive and more reasonable, aligns directly with patients’ beliefs, and is widely accessible. Additionally, herbal remedies are time-tested, considered natural and safer, and are generally believed to have fewer or no side effects, making them an attractive alternative in many healthcare settings (Kumar and Shukla, 2013).

It is concluded by the researchers on the basis of surveys that Herbal and Modern medicinal systems are the two most common systems of medicine used in healthcare (Ahmad and Sharma, 2020; Phondani et al., 2014; Nagori et al., 2011). Traditional/herbal medicines are largely experience-based, but their scientific basis is not satisfactory as compared to modern medicine. Therefore, the transformation from experience-based to evidence-based medicine would be an important step forward.

## Broadband dielectric spectroscopy study

Broadband Dielectric Spectroscopy (BDS) is a versatile and powerful tool for studying molecular dynamics due to its capacity to track molecular mobility across a wide frequency range—up to 16 orders of magnitude. This capability allows BDS to investigate material responses under varying temperatures and pressures, making it a highly valuable method for analyzing the molecular interactions and relaxation behaviours of numerous materials, including pharmaceutical compounds. In pharmaceutical applications, BDS is utilized to assess the molecular dynamics of complex systems such as liquids, liquid crystals, and disordered crystals, as well as to understand charge transport mechanisms in materials like ionic liquids, semiconductors, and ceramics (Woodward, 2021).

The primary mechanism behind dielectric spectroscopy is the application of an external alternating electric field E(ω) to a sample, which induces a response in polar dielectric materials. By examining the response quantified by complex dielectric permittivity $\varepsilon *\left(\omega \right)=\varepsilon^{\prime }\left(\omega \right)-i\varepsilon^{\prime \prime }\left(\omega \right)$ , BDS captures essential data on dielectric dispersion $\varepsilon^{\prime }\left(\omega \right)$ and dielectric absorption $\varepsilon^{\prime \prime }\left(\omega \right)$ . Electrical conduction arising from the translational motions of electric charges (ions, electrons), which can be described by the complex conductivity $\sigma *\left(\omega \right)=\sigma^{\prime }\left(\omega \right)+i\sigma^{\prime \prime }\left(\omega \right)$ (related to the complex permittivity by the equation $\sigma *\left(\omega \right)=i\omega \varepsilon_{0}\varepsilon *\left(\omega \right)$ , where *ε*_0_ is the vacuum permittivity) or the complex electrical modulus $M*\;\left(\omega \right)=M^{\prime }\left(\omega \right)+{\text{iM}}^{\prime \prime }\left(\omega \right)$ related to the complex dielectric permittivity as $\left.\;M*\left(\omega \right)=1∕\varepsilon *\left(\omega \right)\right)$ . These parameters are directly tied to dipole relaxation phenomena, which is due to the reorientational motion of molecular dipoles. This molecular relaxation provides insight into the structural properties of the material and reveals details on phase transitions such as crystallization, vitrification, and chemical reactions, including polymerization, tautomerization, and mutarotation (Wojnarowska et al., 2010). The BDS offers a non-invasive approach to observe and quantify the molecular processes that affect the behaviour and stability of materials, making it particularly useful in pharmaceutical sciences for monitoring drug formulation, stability, and efficacy in response to external electric fields.

## Solubility of drugs and the role of dielectric study in solubility analysis

IUPAC defines solubility as the analytical composition of a saturated solution expressed as a proportion of a designated solute in a designated solvent. Solubility may be stated in units of concentration, molality, mole fraction, mole ratio, and other units (Nic et al., 2005).

Extensive use of solubility from different perspective has led to solubility being expressed in various manners. It is commonly expressed as a concentration, either by mass (g of solute per kg of solvent, g per dL (100 mL) of solvent), molarity, molality, mole fraction, or other similar descriptions of concentration. The maximum equilibrium amount of solute that can dissolve per amount of solvent is the solubility of that solute in that solvent under the specified conditions (Aulton, 2002). The advantage of expressing solubility in this manner is its simplicity, while the disadvantage is that it can strongly depend on the presence of other species in the solvent (e.g., the common ion effect). The Flory-Huggins solution theory is a theoretical framework that describes the solubility behaviour of polymers. In addition, empirical methods like the Hansen Solubility Parameters and Hildebrand Solubility Parameters provide alternative approaches to predict solubility. Solubility predictions can also be derived from other physical constants, such as the enthalpy of fusion. Also, the partition coefficient, commonly represented as Log P, reflects the differential solubility of a compound between a hydrophobic solvent (octanol) and a hydrophilic solvent (water). By taking the logarithmic ratio of these values, compounds can be ranked based on their hydrophilicity or hydrophobicity (Marsac et al., 2006).

The United States Pharmacopeia (USP) and British Pharmacopeia (BP) classify solubility in terms of quantification, regardless of the solvent used, with defined criteria as shown in Table 2. Solubility is a critical factor in drug formulation and bioavailability, particularly for orally administered drugs, where it determines the amount of drug that can be absorbed into systemic circulation. A large portion of newly developed drugs suffer from low water solubility, often resulting in poor bioavailability, which poses challenges for effective therapeutic delivery. Various techniques, such as particle size reduction, crystal engineering, salt formation, and complexation, are employed to enhance drug solubility and dissolution rates, especially for drugs categorized as Biopharmaceutics Classification System (BCS) Class II (low solubility, high permeability) compounds. For these compounds, solubility improvement directly influences bioavailability, making solubility a focal point in pharmaceutical development.

**TABLE 2:** USP and BP solubility criteria [Reproduced from Copyright ^©^ 2012 Savjani et al. (2012)].

Descriptive term	Part of solvent required per part of solute
Very soluble	Less than 1
Freely soluble	From 1 to 10
Soluble	From 10 to 30
Sparingly soluble	From 30 to 100
Slightly soluble	From 100 to 1,000
Very slightly soluble	From 1,000 to 10,000
Practically insoluble	10,000 and over

For solubility classification, Biopharmaceutics Classification System (BCS) uses the highest dose strength of an immediate-release product. A drug is classified as highly soluble if its highest dose strength can dissolve in 250 mL or less of aqueous media within a pH range of 1–7.5. This 250 mL volume reflects standard conditions in bioequivalence studies, where drug products are administered to fasting volunteers with a glass of water. Intestinal permeability classification is determined through comparison with intravenous administration. Therefore, Drugs are categorized into four BCS classes: Class I (high solubility, high permeability), Class II (low solubility, high permeability), Class III (high solubility, low permeability), and Class IV (low solubility, low permeability). Researchers uses various techniques to enhance the solubility of drugs. Among all the solubility enhancement techniques, inclusion complex formation technique has been employed more precisely to improve the aqueous solubility, dissolution rate, and bioavailability of poorly water-soluble drugs (Savjani et al., 2012).

Dielectric spectroscopy helps in selecting excipients that match the dielectric properties of active ingredients. This compatibility prevents precipitation, enhances solubility, and improves bioavailability. For example, functional groups such as O-H and C=C bonds stabilize formulations by promoting hydrogen bonding and dipole interactions. By measuring dielectric constants, researchers can determine solvent compatibility and predict solubility improvements. The principle of “like dissolves like” ensures better formulation strategies by matching polar or nonpolar solvents to the respective compounds. Dielectric studies provide insights into the polarity and interaction mechanisms of drug molecules with solvents, contributing valuable information for solubility enhancement strategies. By measuring a compound’s dielectric constant, researchers can gauge its polarity, which affects its compatibility with different solvents under the principle of “like dissolves like.” This principle implies that polar drugs with high dielectric constants are more soluble in polar solvents, such as water, whereas nonpolar drugs are better suited to nonpolar solvents. Dielectric analysis is also instrumental in studying crystal formation and dissolution, helping scientists assess and optimize the behaviour of drugs in various formulations.

The study on Self-Nanoemulsifying Drug Delivery Systems (SNEDDS) provides valuable insights into how dielectric properties influence drug delivery by enhancing the bioavailability of poorly soluble drugs. Through the use of dielectric spectroscopy, key parameters such as dielectric constant, relaxation time, and dielectric loss factor were measured across various oil concentrations within SNEDDS formulations. This data allowed researchers to analyze the molecular interactions among SNEDDS components, including oil, water, and surfactants. By observing these properties across a GHz frequency range, the study sheds light on molecular orientations and polarization behaviours, particularly the orientation of water molecules around oil droplets, which is critical for ensuring dispersion stability in aqueous environments.

**FIGURE 1:**
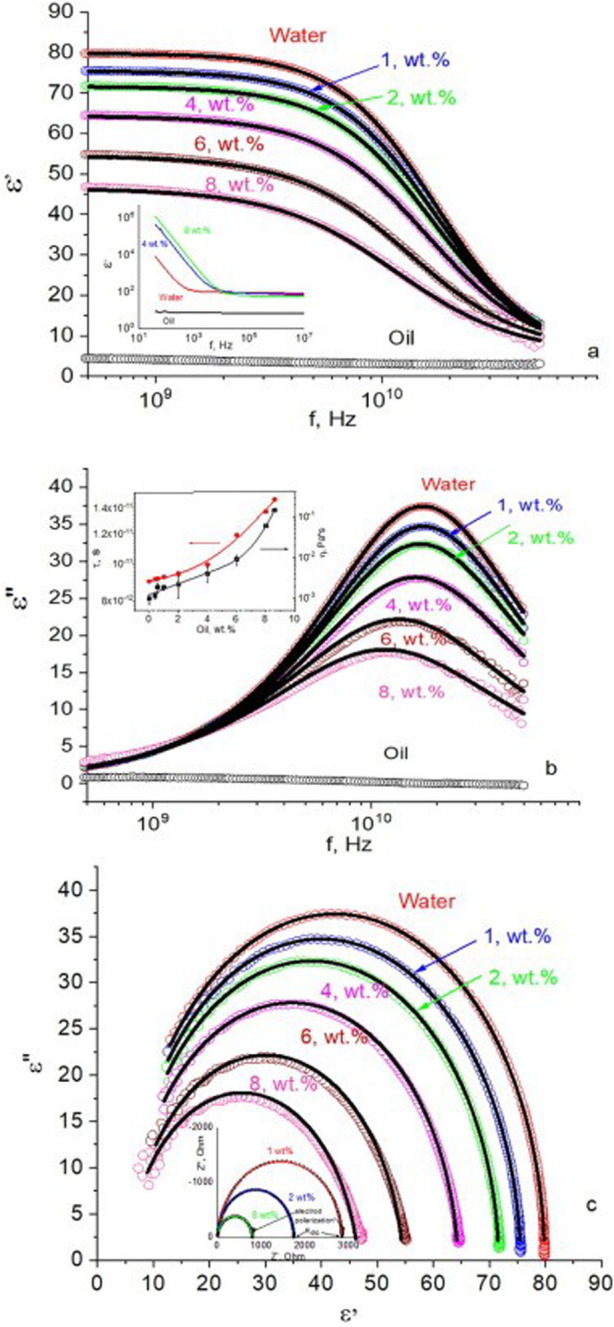
Dependences of **(A)** the real *ε′* **(B)** imaginary *ε″* parts of permittivity on frequency, and **(C)** the Cole-Cole plot obtained in oil–water nano emulsions with oil content indicated on graphs. Open circles are experimental results, continuous lines-results of fitting using cole-cole equation. Inset **(A)** demonstrates the real part of permittivity in the low-frequency range. Inset on **(B)** shows the dependences of relaxation time and viscosity on oil wt. % in the mixture. Inset **(C)** shows impedance spectra in the low frequencies (color online) [Reproduced from ^©^ 2023 Elsevier B.V. All rights reserved] (Alfaro et al., 2023).

One of the primary findings of the study was the relationship between dielectric constant and oil concentration. As oil content in the nano emulsion increased, the dielectric constant decreased due to the inherently lower dielectric properties of oil compared to water as shown in Figure 1A. This trend suggests that dielectric measurements can effectively guide the oil-to-water ratio within SNEDDS, enabling precise optimization of formulations to achieve desired solubility profiles. The dielectric constant’s sensitivity to oil concentration underscores its potential utility in quality control for SNEDDS formulations, ensuring consistency and effectiveness in drug delivery systems.

The study further examined the correlation between relaxation time and viscosity within SNEDDS as shown in Figure 1B. Findings indicated that as oil concentration increased, both relaxation time and viscosity rose, suggesting increased resistance to molecular rotation and dipole reorientation. This relationship aligns with existing literature, which shows that higher viscosity restricts dipolar movement and, consequently, lengthens relaxation time. By understanding this relationship, researchers can tailor SNEDDS formulations to maintain stability and optimize drug encapsulation, ensuring effective and sustained release profiles.

In Figure 1C, the Cole-Cole plot displays the dielectric response measured in oil–water mixtures within the GHz frequency range, with experimental data represented by open points and fitted results by continuous lines. In Figure 1, the various oil weight percentages are displayed. Similar to the permittivity patterns seen in Figures 1A, B, the fitting findings show that the model is accurate because they closely match the experimental measurements. At low frequencies, the inset of Figure 1C further demonstrates how the imaginary part of the impedance depends on the real part. DC adjustment in the equation was made possible by the determination of the DC resistance from this data (as seen in the inset of Figure 1C). Accurately predicting the dielectric behaviour of the emulsion is essential for optimizing its performance in practical applications. The study also identified relaxation peaks and Maxwell-Wagner interfacial polarization (Prodromakis and Papavassiliou, 2009; Dey et al., 2024), revealing shifts in dipole relaxation dynamics due to varying oil concentrations. These results highlight how dielectric properties can be modulated to maintain SNEDDS stability and enhance bioavailability, ultimately contributing to improved efficacy of poorly water-soluble drugs in clinical applications.

## The role of dielectric study in herbal medicines

A solid, quantitative foundation for comprehending herbal medicines’ interactions with solvents, formulation stability, and effectiveness in delivering therapeutic substances is provided by dielectric studies, which provide revolutionary insights into the electrical characteristics of herbal medicines. Herbal formulations often comprise a complex mixture of bioactive constituents, each with distinct dielectric properties that influence their solubility, bioavailability, and interactions with biological systems. By applying dielectric analysis, we can bridge the gap between traditional herbal medicine and advanced scientific methodologies, paving the way for standardized and optimized herbal formulations (Navarrete et al., 2011). Due to the limited literature on the dielectric properties of herbal extracts we particularly focused on the study of Radix Glycyrrhizae extract, examining its dielectric behavior and response to frequency-dependent fields, exemplifies how this analytical approach deepens our comprehension of herbal formulations and their applications. This will enhance the understanding of the role of dielectric study to explore herbal medicines and various other herbal extracts in the future.

**FIGURE 2:**
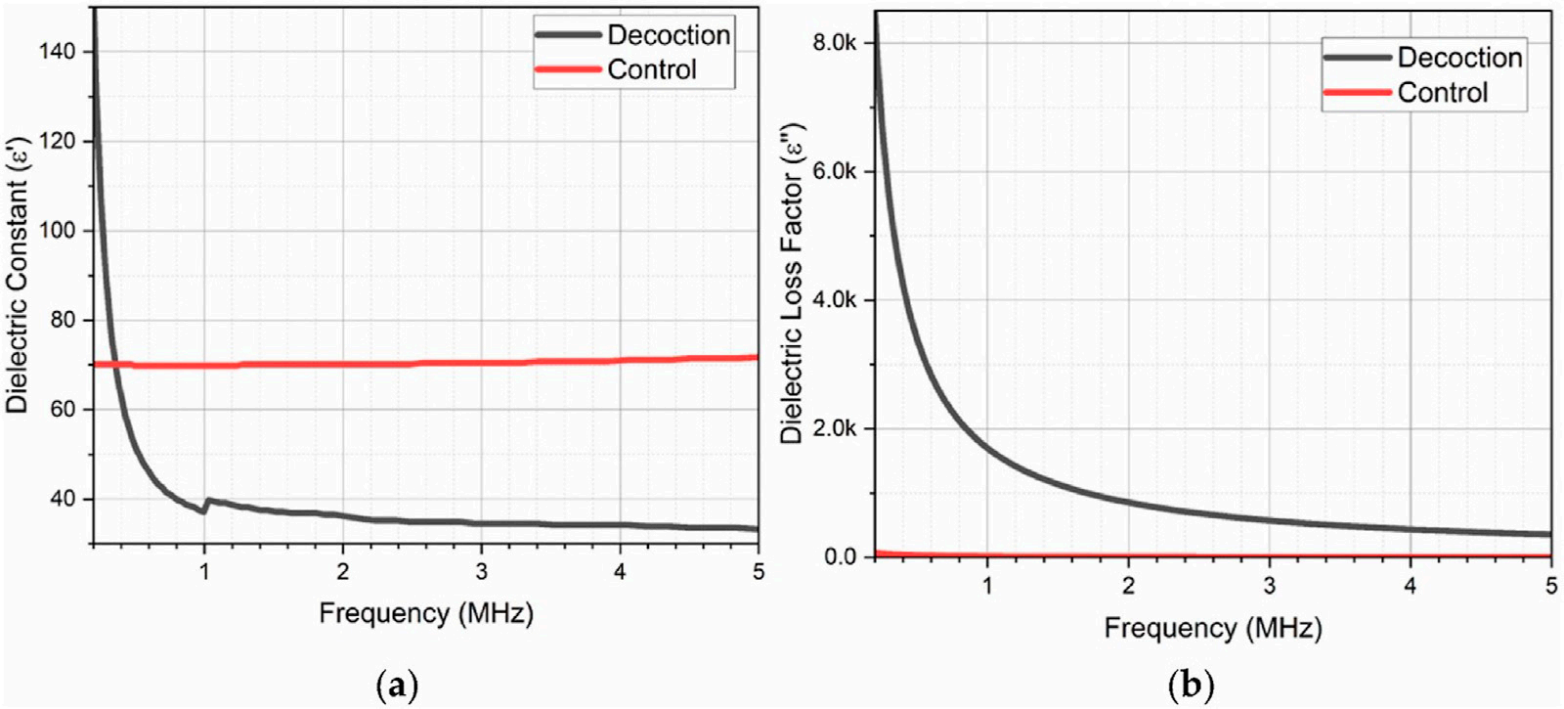
**(A)** Dielectric constant and **(B)** loss factor of Radix Glycyrrhizae decoction extract and control (distilled water) using a Hioki impedance analyzer in the frequency range of 4 Hz–5 MHz [Reproduced from ^©^ 2024 The Authors. Published by Elsevier Ltd. under the CC BY-NC license] (Liang et al., 2024).

A key advantage of dielectric analysis in herbal medicine lies in its predictive power for solubility and solvent compatibility. Dielectric properties, such as the dielectric constant ε′, are indicative of a compound’s polarity and, under the principle of “like dissolves like,” help determine compatibility with various solvents as discussed earlier. Compounds with high dielectric constants tend to dissolve better in polar solvents, making aqueous extractions ideal for polar bioactives. The high dielectric constant observed for Radix Glycyrrhizae at low frequencies reflects its strong polarity and its effective extraction in water—a common preparation in traditional medicine, as demonstrated in Figure 2. This intrinsic polarity of Radix Glycyrrhizae renders it well-suited for water-based formulations, enhancing the solubility and extractability of its bioactive constituents.

In Figure 2A, the dielectric constant of the Radix Glycyrrhizae extract exhibits a marked response at lower frequencies, with an elevated value around 4 Hz indicating strong polarization. As the frequency increases, the dielectric constant progressively declines, signifying a reduction in polarization, until it stabilizes beyond 1 MHz, showing only minor fluctuations up to 5 MHz, which is primarily due to molecular dipole relaxation. Polar covalent bonds, particularly those involving oxygen (such as hydroxyl groups in glycyrrhizins and other polar groups within glycosides and glycyrrhetinic acid), are central to the dipole polarization observed in the extract. As frequency increases, these polar molecules lack sufficient time to reorient in sync with the rapidly oscillating electric field, resulting in a reduction in polarization effects. Additionally, ionic metabolites and electrolytes within the extract exhibit limited mobility at higher frequencies, as their oscillations increasingly fall out of phase with the electric field frequency, further diminishing polarization. This frequency-dependent dielectric behavior highlights the inherent polarization characteristics of the extract’s bioactive compounds (Liang et al., 2024). Distilled water was utilized as a control for comparison of dielectric constant and loss factor. Unlike the decoction extract, distilled water displayed minimal frequency-dependent variation, underscoring that the dielectric behavior of the Radix Glycyrrhizae extract is not predominantly influenced by its aqueous component. This suggests that the bioactive constituents within the extract are the primary drivers of its distinctive dielectric profile.

The loss factor of the Radix Glycyrrhizae extract is shown in Figure 2B, which is notably higher at lower frequencies and diminishes with increasing frequency. This trend reaches a minimum around 5 MHz, further emphasizing the frequency-dependent characteristics of the extract. This attributed to reduced energy dissipation during polarization. This reduction is due to diminished friction among polar phytochemical groups—such as hydroxyl, carboxylic acid, carbonyl, and ester groups—with increasing frequency. At higher frequencies, the molecular interactions that typically contribute to energy dissipation become limited, leading to lower loss factors. These frequency-dependent dielectric properties underscore the dynamic behavior of bioactive constituents in the Radix Glycyrrhizae extract and offer valuable insights into the extract’s molecular interactions, which are crucial for understanding its formulation stability and bioavailability potential. These findings collectively suggest that the dielectric properties of the Radix Glycyrrhizae extract are largely governed by the molecular composition and dynamic responses of its bioactive ingredients, providing a deeper understanding of its formulation stability and potential applications in therapeutic delivery (Ruan et al., 2016).

Dielectric analysis also plays a critical role in improving formulation stability. In pharmaceutical formulations, selecting excipients that closely match the dielectric properties of active ingredients can prevent precipitation, enhance solubility, and increase bioavailability. The Radix Glycyrrhizae study highlights this by showing how functional groups like O-H and C=C bonds influence dielectric behaviours. These groups promote hydrogen bonding and dipole interactions that stabilize the extract in solution, reducing degradation risks. With a clear understanding of dielectric constants and loss factors, formulators can select co-solvents and stabilizing agents that harmonize with the active compounds, yielding formulations with greater consistency and therapeutic efficacy. The study also links the antioxidant properties of Radix Glycyrrhizae with dielectric loss, observing a concentration-dependent increase in radical scavenging activity. Dielectric loss at higher frequencies implies that these bioactive compounds contribute both antioxidant effects and energy dissipation, which can be advantageous for formulations requiring thermal management.

**FIGURE 3:**
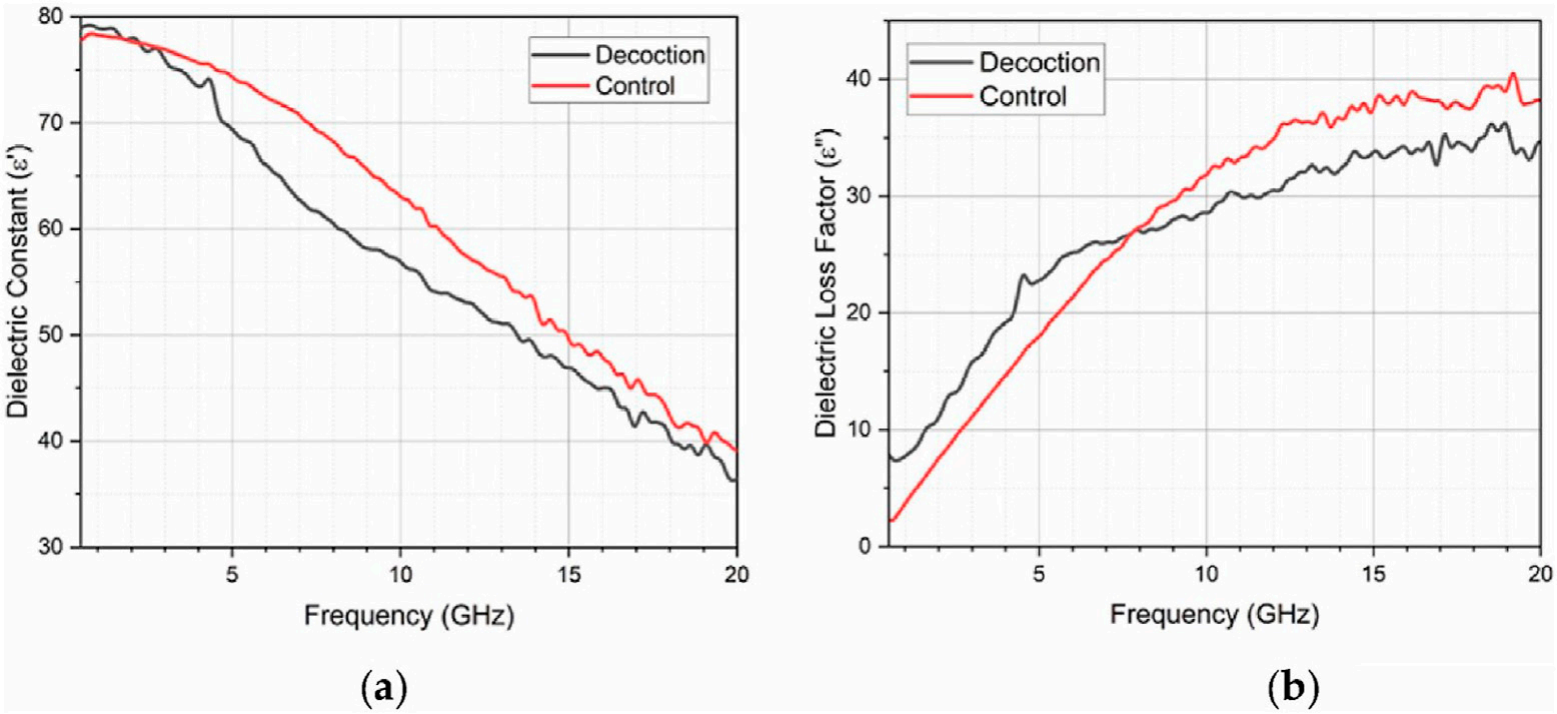
Comparative study of the dielectric properties of Radix Glycyrrhizae decoction and a control using vector network analyzer within the frequency range of 5 MHz–20 GHz. **(A)** Fluctuation of the dielectric constant with frequency. **(B)** Fluctuation of the dielectric loss factor with frequency [Reproduced from ^©^ 2024 The Authors. Published by Elsevier Ltd. under the CC BY-NC license] (Liang et al., 2024).

The dielectric spectrum contrasts between 4 Hz and 50 MHz and 50 MHz to 20 GHz, where the dielectric loss factor increases in the later range. The mobility of ionic metabolites, electrolytes, and minerals within the extract decreases. This leads to reduced ionic conductivity and a decline in dielectric constant as shown in Figure 3A. Meanwhile, the loss factor increases due to intensified frictional activities during ion oscillations at higher frequencies compared to lower frequencies as shown in Figure 3B. Moreover, hindered response of water molecules to electric fields contributes to decreased dielectric constant at higher frequencies. While increased loss factor results from damping of molecular motions. Both dielectric constant and loss factor show minimal differences between the decoction extract and distilled water. This is due to water molecules, which is the major component of the decoction. The dielectric properties of Radix Glycyrrhizae decoctions, including dielectric constant and loss factor, significantly influence their therapeutic potential. The dielectric constant, which measures energy storage in an electric field, is affected by concentrations and types of phytoconstituents such as glycyrrhizin, altering interactions with tissues. Interactions between phytoconstituents alter the dielectric loss factor, which measures electromagnetic energy dissipation and may improve therapeutic benefits. The phenolic contents, antioxidant activity, and herbal yield of purslane (*Portulaca oleracea* L.) cultivated on various soilless media in a closed system. Dielectric characteristics are also influenced by moisture content, which is important in aqueous decoctions.

Dielectric characteristics of aromatic plants are measured and estimated. Application to rosemary with low moisture content Simultaneously, a dielectric analysis revealed strong polarization processes impacted by flavonoids and phenols. Improved dielectric responses are correlated with higher TPC and TFC. In the range of 5 MHz–20 GHz, analyzed with a vector network analyzer, polarization decreases with frequency, while the loss factor increases, reflecting effective energy dissipation. These findings are supported by FTIR, identifying molecular vibrations affecting dielectric behaviour. Antioxidant activity, assessed via the DPPH assay, further impacts dielectric properties, suggesting stable electrical performance alongside antioxidant benefits. This integrated approach informs potential applications in pharmaceuticals, biomedicine, and food science, highlighting the critical role of both electrical and bioactive properties in Radix Glycyrrhizae decoctions (Liang et al., 2024).

## Crystallization kinetics of drugs

In the study of crystallization kinetics in amorphous drugs, dielectric analysis serves as a critical tool for understanding molecular mobility and phase transitions. Using Dynamic Dielectric Spectroscopy (DDS) and Thermostimulated Current (TSC) techniques, researchers can observe the crystallization of amorphous pharmaceutical compounds in real time. In this particular study of the SSR drug, dielectric analysis was employed to examine the transition from amorphous to crystalline states over a range of temperatures. By monitoring changes in dielectric response, the researchers could identify two primary relaxation processes: the α-relaxation, associated with cooperative molecular motions near the glass transition temperature (*T*g), and the β-relaxation, which represents intramolecular oscillations of small dipolar groups. Findings revealed that the β-relaxation mode predominantly influences the crystallization rate, as its Arrhenius temperature dependence enabled efficient crystal growth within the amorphous matrix (Alie et al., 2004).

**FIGURE 4:**
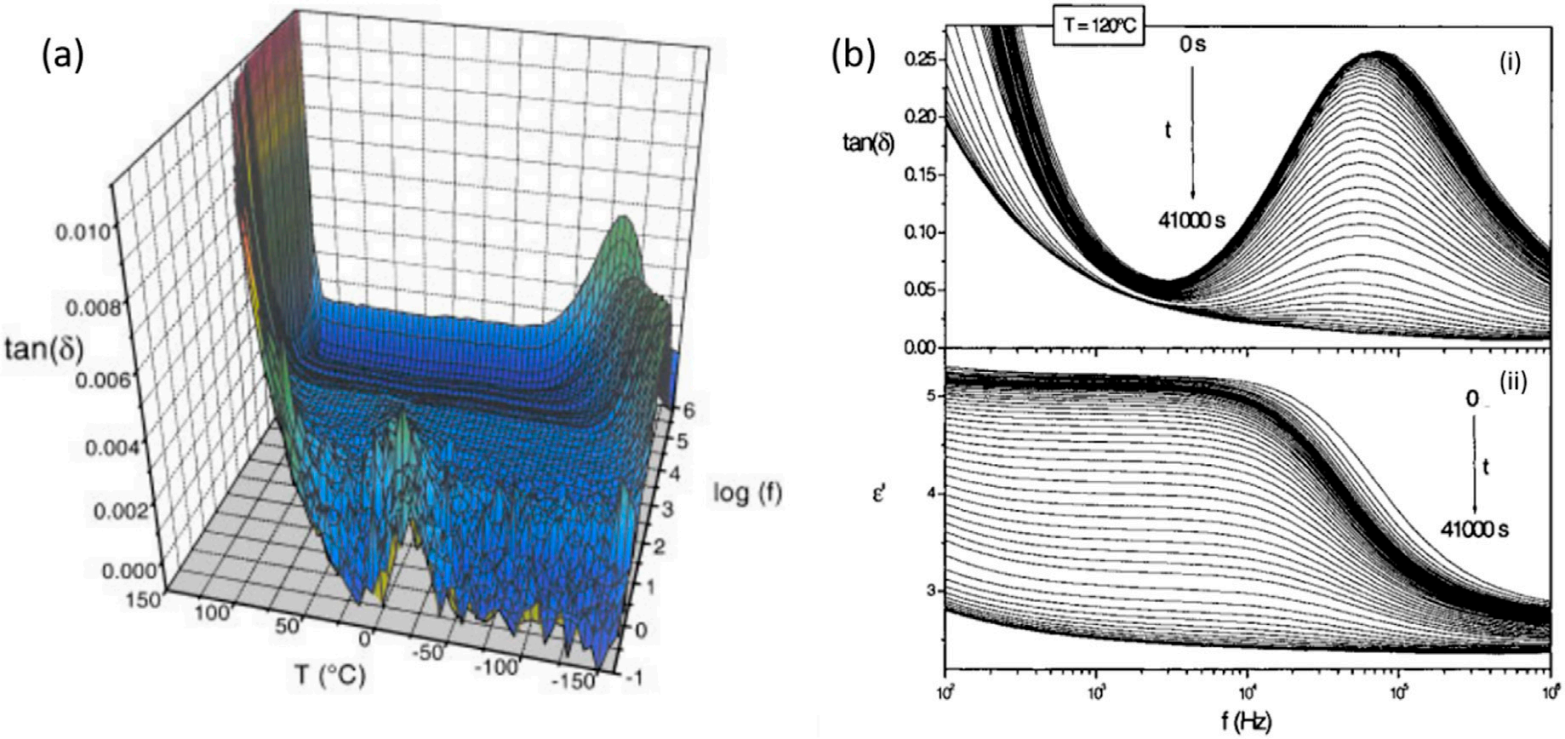
**(A)** The dielectric loss factor, tan δ for SSR in the A crystalline form is presented as a function of temperature and frequency. **(B)** Frequency-dependent isothermal DDS measurements at 120°C over different time intervals, showing: (i) the loss factor, tan δ and (ii) the real part of the complex permittivity, ε′ Arrows on both figures indicate a decreasing signal with increasing time [Reproduced from Copyright ^©^ 2004 Wiley-Liss, Inc. Published by Elsevier Inc. All rights reserved] (Alie et al., 2004).

This real-time analysis, facilitated by dielectric spectroscopy, allowed the researchers to map the crystallization kinetics by tracking the decrease in dielectric response over time as crystallization proceeded. Through the Kohlrausch–Williams–Watts (KWW) model, the degree of crystallinity was quantitatively measured, showing that the crystallization process follows a two-phase model where amorphous and crystalline phases coexist. Interestingly, the dielectric strength decrease followed an Arrhenius model, indicating that crystallization accelerates with temperature. Further, the study noted that crystallization resulted in different crystalline forms (A and B) depending on the temperature, with form B favoured at higher temperatures (above 120°C) and form A at lower temperatures. This detailed dielectric study demonstrates how molecular mobility can dictate the crystallization process, impacting stability and storage considerations for pharmaceutical formulations. Figure 4A demonstrates that the frequency-temperature range of the dielectric relaxation mode detected for the A crystalline form of SSR does not overlap with the range of the α-relaxation mode observed in the amorphous form. This supports the use of the α-relaxation mode to monitor the crystallization of the amorphous SSR and confirms that the dielectric responses of crystalline and amorphous phases do not interfere. Figure 4B illustrates the isothermal crystallization of amorphous SSR at 120°C over time, showing a reduction in the intensity of the loss peak and the real part of the complex permittivity associated with the α-relaxation mode. These changes confirm the progression of crystallization during the experiment.

The study highlights that the dielectric relaxation mode in the A crystalline form of SSR lies outside the frequency–temperature range of the α-relaxation mode, confirming the latter’s relevance for tracking the crystallization of the amorphous SSR. Real-time dielectric measurements in the 100°C–140°C range showed no interference between crystalline and amorphous dielectric responses. Isothermal cold crystallization experiments revealed a decrease in the intensity of the loss peak and the real part of complex permittivity associated with the α-relaxation mode over time, indicating crystallization of the amorphous SSR.

**FIGURE 5:**
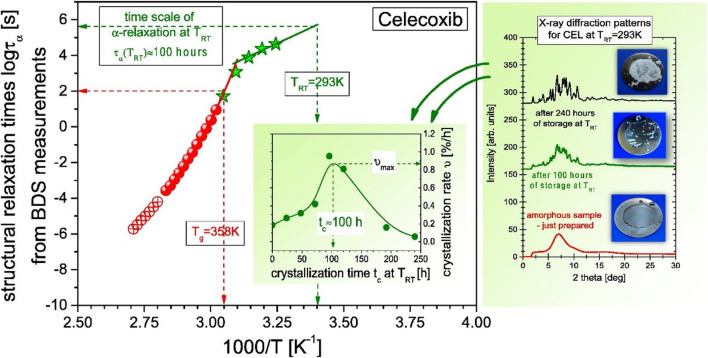
Plot of relaxation time against crystallization time to understand properties of molecular dynamics and crystallization process [Reproduced from Copyright ^©^ 2015 Elsevier B.V. All rights reserved] (Grzybowska et al., 2016).

The analysis of relaxation processes in glassy and supercooled liquid states of amorphous API with molecular dynamics distributed over many decades in frequency, becomes a powerful tool for addressing many critical problems related to their successfully application. The pharmaceuticals can be thoroughly examined by means of the broadband dielectric spectroscopy, which is a very useful experimental technique to explore different relaxation processes, stability assessment and crystallization kinetics shown in Figure 5 (Knapik-Kowalczuk et al., 2021; Grzybowska et al., 2016). The studies can be conducted under different temperature and pressure ranges. The obtained data needed in probing correlations between properties of molecular dynamics and crystallization process. This data may assist developing effective and efficient methods for stabilizing amorphous drugs.

The dielectric measurements were performed according to the time domain spectroscopy principle covering the frequency interval 6.7 MHz to 5 GHz. Pilocarpine chloride interacts with the colloid structures as an electrolyte, while chloramphenicol seems to be located in the interfacial (w/o) membrane (Skodvin et al., 1993). Microwave-based approaches offer a fast and cost-effective way to detect quality variations, providing an alternative to traditional techniques in the pharmaceutical and cosmetic industries (Zaarour et al., 2023).

## Drug-herb interactions

The pharmacodynamic mechanism of drug-herb interactions involves the modulation of drug effects by active compounds in herbs through interactions at drug targets such as receptors, enzymes, and ion channels. These interactions may enhance or diminish therapeutic efficacy or cause adverse effects. Understanding these mechanisms requires integrating diverse scientific techniques, including cutting-edge approaches like dielectric spectroscopy, molecular assays, imaging technologies, and pharmacovigilance, to characterize these interactions at various levels.

### Mechanistic studies using receptor binding assays and dielectric spectroscopy

Receptor binding assays have traditionally provided insights into agonistic and antagonistic herb-drug interactions. For example, St. John’s Wort (*Hypericum perforatum*) inhibits serotonin reuptake and modulates serotonin receptors. This leads to enhanced effects of selective serotonin reuptake inhibitors (SSRIs). Radioligand binding techniques and competitive assays reveal how herbal compounds compete with drugs for receptor binding. This helps in highlighting the risks of serotonin syndrome (Müller et al., 1998). In the similar way, dielectric spectroscopy has been used to investigate the interaction of phytochemicals like flavonoids with membrane receptors or ion channels. This investigation reveal changes in membrane polarization and drug efficacy (Bakhtiari et al., 2022). It provides a non-invasive and quantitative method to assess how herbal compounds alter the microenvironment around drug targets.

### Enzyme activity assays and modulation by herbs

The enzymatic activity is often modulated which affect drug action and metabolism. Biochemical assays quantifying enzyme inhibition or activation provide key data for understanding these interactions. Considering the case of Curcuma longa (turmeric), which inhibits cyclooxygenase-2 (COX-2) and potentiating the anti-inflammatory effects of NSAIDs. Using spectrophotometric and chromatographic techniques helps in enzyme kinetics studies and elucidated inhibitory constants (Ki) and synergistic interactions (Jurenka, 2009). Dielectric spectroscopy complements these studies by assessing conformational changes in enzymes upon herb binding. For example, the interaction of polyphenols from green tea with acetylcholinesterase has been analyzed using dielectric techniques. This showed shifts in enzyme relaxation frequencies corresponding to structural alterations. This approach can provide a real-time assessment of enzyme-herb interactions, particularly for drug-metabolizing enzymes like cytochrome P450 isoforms (CYP450).

### Cellular and molecular-level interactions

Cell culture models and molecular assays are vital for studying herb-drug interactions at the cellular level. For instance, co-incubation of cancer cell lines with Ephedra sinica extracts and chemotherapeutics enhances apoptosis through caspase activation. Techniques like flow cytometry and Western blotting reveal upregulation of apoptotic markers such as cleaved caspase-3 and PARP (Li, 2020). Dielectric spectroscopy applied to cell systems enables monitoring of cellular responses to drug-herb combinations in real-time. Changes in the dielectric constant of cell suspensions, due to alterations in cell membrane integrity or intracellular signalling, provide quantitative data on cytotoxicity or synergistic effects. This is particularly useful in studying immune-modulating herbs like Echinacea, which interact with immunosuppressants by altering cytokine expression profiles (Gertsch et al., 2004).

### Neuropharmacological insights

Herbs with central nervous system activity often interact with psychotropic drugs. For example, Valeriana officinalis enhances GABAergic signalling, potentiating the effects of benzodiazepines and increasing sedation risks (Spinella, 2001). Patch-clamp electrophysiology demonstrates how herbal extracts modulate ion channel activity, but dielectric spectroscopy offers a complementary approach by analyzing changes in ionic conductivity and polarization across neural membranes.

### *In vivo* and imaging techniques

Animal models remain central to studying systemic effects of herb-drug interactions. Rodent studies have shown that Panax ginseng enhances anticoagulant effects when combined with warfarin, assessed through bleeding time and clotting factor assays. Imaging technologies like MRI and PET allow real-time tracking of herb-drug effects *in vivo*. For instance, radiolabeled glucose analogs have demonstrated that ginseng enhances glucose uptake in the brain, which could modulate the pharmacodynamics of antidiabetic drugs (Zhuang et al., 2021). Dielectric spectroscopy is now being explored *in vivo* to study tissue dielectric properties in response to herb-drug combinations. For example, differences in tissue permittivity before and after herbal supplementation can reveal alterations in drug distribution and metabolism.

Pharmacovigilance systems, coupled with data mining and machine learning, identify real-world patterns of adverse herb-drug interactions. Dielectric spectroscopy can further refine these findings by enabling rapid, label-free screening of complex herb-drug mixtures, bridging the gap between clinical observations and mechanistic studies. While dielectric spectroscopy offers significant advantages in studying drug-herb interactions, challenges remain in correlating dielectric properties with specific molecular or cellular mechanisms. Advanced computational modelling and integration with techniques like mass spectrometry and NMR spectroscopy can overcome these limitations, offering a holistic understanding of pharmacodynamic interactions. The pharmacodynamic mechanism of drug-herb interactions spans molecular, cellular, and systemic levels, with scientific techniques like receptor binding assays, enzymatic studies, molecular assays, imaging technologies, and dielectric spectroscopy offering crucial insights. Dielectric spectroscopy, in particular, is a promising tool for real-time, non-invasive studies of herb-drug interactions, providing a novel dimension to this field. As the use of herbal remedies alongside pharmaceuticals grows, a multidisciplinary approach is essential to predict, mitigate, and manage these interactions effectively.

## Conclusion

Although, the allopathic Medicare is the youngest of all systems of medications, it remains to be the most preferred choice of the world today because of its credible evidence based scientific system of healthcare. Despite the benefits of allopathic medicine, there is a growing shift towards herbal medicine. Being cost-effective and easily accessible herbal medicines are utilised very frequently. Rigorous scientific research is the need of the hour to understand the interactions of herbal drugs with conventional drugs. The main constraint of modern allopathic system is that many of its drugs have side effects that could be life threatening, particularly in case of complex diseases such as cancers, AIDS, geriatric, and cardio vascular diseases where prolonged multi-drug therapies are required. Solution to the current problems lies in innovations, that is, exploration of traditional systems of medicines which could serve as complementary or alternative to the allopathic. Quite recently, synergistic combination of herbal formulations with allopathic drugs has been identified as a key area of future research. Several medicinal herbal formulations have been clinically validated for counter-indications, side effects, and toxicities in the line of allopathic medicines. Herbal formulations deserve to be the most efficacious of all traditional systems of medications for synergistic amalgamation with allopathic drugs in amelioration of side effects of the later.

This accentuates the scientific advancements that can aid in understanding these interactions. Studies on dielectric properties and nanodielectric materials contribute to the development of more precise and effective medical devices, enhancing the overall treatment landscape. Dielectric properties in terms of dielectric constant, dielectric loss and loss tangent can be studied to understand the interactions between different formulations. In conclusion, the cogent investigation of herbal extracts with modern pharmaceuticals hold significant promise for advancing medical treatment. By bridging the gap between traditional and modern medicine, this integrative approach can lead to innovative and effective healthcare solutions.

## Future scope

Dielectric spectroscopic study plays a vital role for material characterization and is used to study inter or intra molecular interactions as well as molecular structures of system. The range of potential applications of dielectric spectroscopy is quite broad. The dielectric properties greatly aid in the investigation of the molecular structure and dynamics of condensed matter. These properties help in gathering the information regarding the molecular structure, shape and size etc., of the material. Permittivity of material reflects the ability of the material to get polarized with applied electric field. When the material is kept in an electric field, the material will get polarized due to the induced dipole moment. This is due to the motion of charged atoms/molecules due to the effect of the applied electric field. Polarization is a purely frequency dependent phenomena which may get affected due to the size of molecule and temperature. The relaxation time of herbal material is related to the size of the molecule and the mobility of molecules in liquid.

Neem and Papaya leaf extracts offer significant medicinal properties, including anti-inflammatory, antioxidant, and immunomodulatory effects, which can enhance the therapeutic efficacy of Paracetamol while potentially mitigating its hepatotoxic side effects. Thus, the aim is to study these medicines to monitor multiple physical properties of complex structures. The obtained results can be commendably used in various fields like in designing, development and synthesis of drugs. Dielectric spectroscopy, as a powerful analytical tool, will play a crucial role in this integrative approach.
